# Maclise’ Surgical Anatomy

**Published:** 1850-09

**Authors:** 


### ARTICLE III.

*Surgical Anatomy.*—By JOSEPH MACLISE, Surgeon. With Colored Plates. Phila.: Lea & Blanchard, 1850. Imperial quarto. Part II. (From the Publishers.)

We have received the second part of this magnificent work, containing the surgical anatomy of the hand, the organs of the thorax, abdomen, and the hernial regions. After what we have already said of this work, we have nothing more to add, except that this part is quite equal to the other in exactness of delineation, and beauty of typographical execution. It should be on every surgeon's table.

